# Wind Booster Optimization for On-Site Energy Generation Using Vertical-Axis Wind Turbines

**DOI:** 10.3390/s21144775

**Published:** 2021-07-13

**Authors:** Marco A. Moreno-Armendáriz, Carlos A. Duchanoy, Hiram Calvo, Eddy Ibarra-Ontiveros, Jesua S. Salcedo-Castañeda, Michel Ayala-Canseco, Damián García

**Affiliations:** 1 Instituto Politécnico Nacional, Centro de Investigación en Computación, Av. Juan de Dios Bátiz s/n, Ciudad de México 07738, Mexico; duchduchanoy@cic.ipn.mx (C.A.D.); hcalvo@cic.ipn.mx (H.C.); gabriel040595@gmail.com (E.I.-O.); 2 Cátedra CONACyT, Instituto Politécnico Nacional, Centro de Investigación en Computación, Av. Juan de Dios Bátiz s/n, Ciudad de México 07738, Mexico; 3 Instituto Politécnico Nacional, Unidad Profesional Interdisciplinaria en Ingeniería y Tecnologías Avanzadas, Av Instituto Politécnico Nacional No. 2580, La Laguna Ticoman, Gustavo A. Madero, Ciudad de México 07340, Mexico; jesua.salcedo@gmail.com (J.S.S.-C.); michelayala4@gmail.com (M.A.-C.); 4 Escuela Superior de Cómputo, Instituto Politécnico Nacional, Av. Juan de Dios Bátiz s/n, Col. Lindavista, Ciudad de México 07738, Mexico; damiangarcia.g95@gmail.com

**Keywords:** green energy, Vertical Axis Wind Turbine (VAWT), omni-direction-guide-vane (ODGV)

## Abstract

Large cities have a significant area of buildings with roofs that are not used most of the time. Vertical-axis wind turbines are suitable for this kind of on-site renewable energy generation. Since wind speeds are not high in these cities, a suitable solution to improve energy generation is to add a Wind Booster. This paper presents a methodology useful for selecting and optimizing the main components of a Wind Booster. As a case of study, we present this methodology in a Wind Booster for a Vertical Axis Wind Turbine (VAWT) that considers the wind flow’s specific behavior in a particular city. The final Wind Booster design is state of the art and makes use of Computational Fluid Dynamics (CFD) and Design of Experiments (DOE) techniques. We experimented with the conditions of Mexico City, obtaining a 35.23% increase in torque with the optimized Wind Booster configuration. The results obtained show the potential of this methodology to improve the performance of this kind of system. Moreover, since wind behavior is very different in each city, our proposal could be beneficial for researchers looking to implement the best possible wind turbine in their locality.

## 1. Introduction

Renewable energy generation is becoming an issue of crucial importance, due to the increase in pollution from the use of fossil fuels, stemming from the first industrial revolution, causing an increase in the demand for renewable energy. Some examples of this type of energy are solar, hydraulic, geothermal, and wind energy. Wind power is one of the renewable energy sources with the most potential [1] due to its high profitability and performance. The installed wind capacity worldwide is 650.8 gigawatts, representing 6% of the electricity generated, according to statistics published at the end of 2019 by the World Wind Energy Association (WWEA) [2].

Several countries continue to satisfy their energy supply mainly with fossil fuels, gas and oil being the most frequent sources. For example, in Mexico, the most recent energy balance reports that hydrocarbons contributed to 70.5% of their primary energy production in 2017. The use of renewable energy has made slow progress. In 2018, the implementation of these energies increased by 14.1%; as a result, 29.5% of the total production of electrical energy in Mexico was from renewable energy. With the recent changes made to the energy reform by 2024, the percentage of this kind of energy is expected to rise to 35% [3].

Among the different types of renewable energy, wind power has spread worldwide since it is possible to obtain it in various latitudes of the planet, as shown by the extensive wind farms located in the mountains or near the coast. However, urban areas are a place in which this kind of energy is still not entirely exploited, although there exists the possibility of capturing the surrounding air masses.

Extracting energy from renewable sources close to populated areas where power is required is known as *on-site renewable energy generation* [4]. This approach has been studied in several works [5,6,7,8,9,10]. In [11], the authors mention the following main advantages: (1) exploitation of resources in areas not suitable for large centralized generation stations; (2) significant decrease in losses associated with power transmission and distribution; (3) economic viability [12]; and (4) lower vulnerability in the case of a natural disaster or a terrorist attack. Notwithstanding these benefits, there are some challenges to overcome such as (1) technical issues [13,14], (2) regulatory barriers [13], (3) added difficulties in maintenance and operations due to an enormous number of stations, and (4) environmental issues [13]. Another interesting work is [10], where the authors presented a novel tool for assessing solar radiation (SOLIS).

According to their orientation and axis of rotation, the main types of wind turbines are Horizontal Axis Wind Turbines (HAWTs) and Vertical Axis Wind Turbines (VAWTs) [15]. On the one hand, HAWT requires high wind speeds to start, depends on wind direction, and needs laminar wind. On the other hand, VAWT starts at low wind speeds, it does not depend on wind direction, it can work with the presence of turbulent wind, and the construction is cheap. All these features make VAWT more suitable for use in large cities, as in our case study.

HAWTs are the most common and efficient method since they exploit more wind energy due to the design of their blades (from 1 to 3). However, they require high wind speeds, a strong tower to support the weight of the gondola, and the installation cost is higher, in addition to requiring orientation systems since the forces do not tend to guide it naturally. Thus, they use orientation tails in the case of small turbines, and orientation servomechanisms in the case of large ones. On the contrary [16], VAWTs have the advantage of not needing orientation systems and having lower cost of construction, installation and easier maintenance, low noise and angular velocity in operation, reduced wear on moving parts, various rotor configuration options, and high static and dynamic moments.

### 1.1. Aim of the Study

This work focuses on finding a way to improve the green energy produced by a small VAWT suitable for installing on the roofs of houses and buildings in Mexico City. To this end, first, we selected a VAWT and then we reviewed the state of the art of Wind Boosters (WBs) for the VAWT. Finally, we proposed our own WB, and we optimized its performance.

### 1.2. Savonius Wind Turbine

The Savonius wind turbine, invented by Finnish engineer Sigurd Johannes Savonius in 1922 [17], is one of the simplest turbines, as it is a drag force wind turbine consisting of two or more blades attached to a central axis in opposite directions. The aerodynamic efficiency of this rotor is lower than other types; however, it is robust, has an excellent starting torque efficiency, and operates with less dependency on wind direction. Nowadays, small wind energy is one of the most exciting alternatives for homeowners that are looking to reduce their electricity bills and obtain self-produced energy. Accordingly, the Savonius rotor is one of the most studied in order to enhance its performance.

A significant drawback of the Savonius rotor is that a negative torque appears during the rotational cycle of the rotor, so the total positive torque decreases. One way to enhance its performance is to test the design of various blade profiles. The most common are Semicircular [18], Twisted [19], Bach [20], Modified Bach [21], Elliptical [22], Roy profile [21], and Spline [23]. Some interesting patents are [24,25].

### 1.3. Wind Booster

Even though the Savonius has essential characteristics necessary for its use in cities and low wind areas, it also has deficiencies, which make this type of wind turbine only used to satisfy limited electricity consumption needs. On the path to achieving usage of these kinds of generators as a real option for generating electricity, an affordable option is the use of augmentation techniques. An augmenter aims to concentrate the wind flow before it arrives at the rotor [26]; this is very helpful since the power produced by the turbine is proportional to the cube of the incoming wind speed. So a mild increase in the wind speed boosts the rotor efficiency. Furthermore, since the Betz limit is 59.3% [15,27], an augmentation system can be used to surpass this value. The devices used for this task can be a stator, diffuser, or guide vanes, among others.

#### 1.3.1. Single Direction Flow Inlet

This kind of augmentation device consists of one or more stators placed on the upwind side of the turbine. It can be a straight plate, curved plate, or any other shape. Mostly, these plates help to reduce the negative torque generated on the VAWT to lead the flow to a better angle of attack, or to concentrate the wind flow to raise its velocity. Some exciting works in this area are Refs. [28,29,30,31].

#### 1.3.2. Omni-Direction Flow Inlet

**Figure 1:**
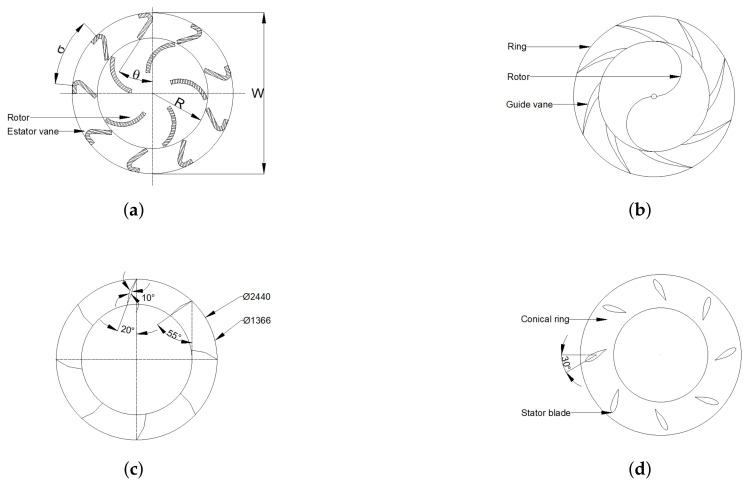
Different omni-directional wind boosters. (**a**) Geometrical variables of Zephyr VAWT. (**b**) Korprasertsak et al. Savonius with wind booster. (**c**) Wong et al. ODGV design. (**d**) Nobile et al. 3D stator.

The augmentation systems of Section 1.3.1 are useful only for single direction wind flow, so they need a yaw mechanism. Considering that wind flow comes from any direction and changes all times, some researchers proposed omni-directional augmentation systems to overcome this restriction, similar to [32], where Pope et al. presented a new drag-type wind turbine called Zephyr VAWT, as shown in Figure 1a. With the stator vanes, the $C_{P}$ of the turbine alone (ratio between turbine’s generated power and available wind’s power) increases from 0.098 to 0.12; nonetheless, it is still a low $C_{P}$ value. Korprasertsak and Leephakpreeda [33] analyzed the impact of a wind booster on a drag-type Savonius VAWT. As shown in Figure 1b, the booster consists of upper and lower rings with stator vanes equally placed around the Savonius; this allows it to capture wind flow from all directions. With this device, the simulations showed a 50% increase in the rotational speed under a no-load condition. In [34], Wong et al. presented an omni-direction-guide-vane (ODGV) where each of the guide vanes was divided into two equal slices and bent at an angle of $10^{\circ }$ as shown in Figure 1c. Simulations showed an enhancement of 31.65% concerning the original ODGV. Nobile et al. [35] developed a similar design, where an ODVT was installed around an H-rotor VAWT, as shown in Figure 1d. The stator has two conical surfaces and eight straight vertical blades. The outer edges of the tapered surfaces promote turbulent mixing above and below, reducing the backpressure inside the stator in such a way that it increases the power output of the turbine. The simulations showed a rise near 30–35% in $C_{P}$ and the ratio between the turbine’s torque and the wind’s available torque ($C_{T}$). It is important to note that these studies were performed using CFD simulations.

## 2. Methodology

**Figure 2:**
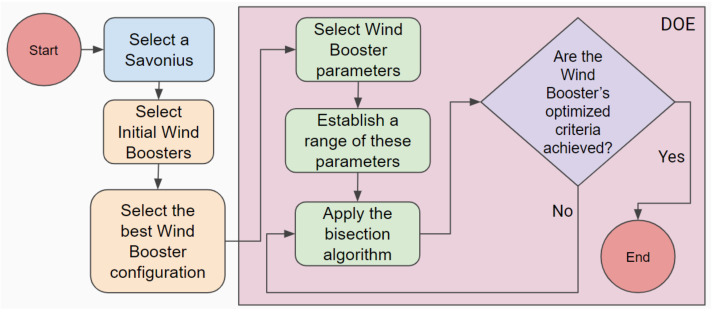
Proposed Methodology with Design of Experiments (DOE) on the right side.

The central idea of this work is the methodology shown in Figure 2. As an example, we focus on the optimization of the Wind Booster. To our knowledge, this is the first time a methodology for this purpose is proposed. Additionally, note that the same process could optimize Savonius or other elements of this kind of system. In the next subsections, we explain each step in detail.

### 2.1. Select Savonius

**Figure 3:**
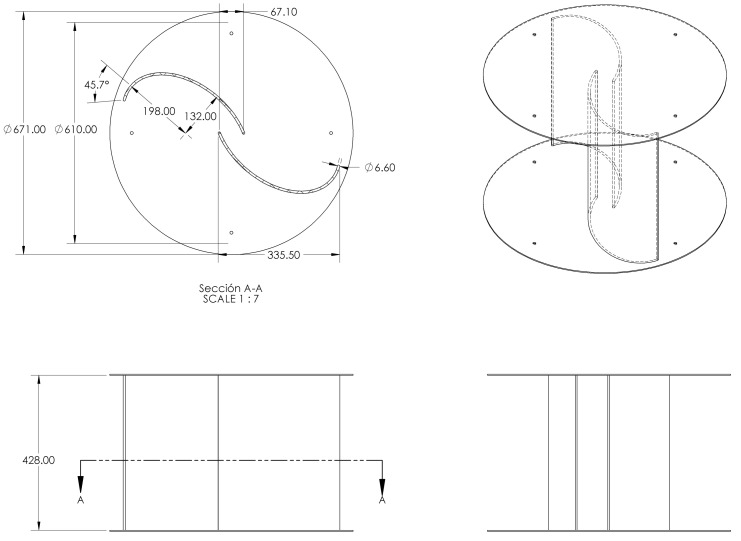
Savonius final design.

A common way to compare the performance of the Savonius rotors is by calculating the torque coefficient ($C_{t}$) and power coefficient ($C_{p}$) [36]. In [37], the authors presented a comparison table of different blade profiles, each one with its corresponding $C_{Pmax}$. From this table, the Elliptical [22] reports $C_{Pmax}$ = 0.33; therefore, since we only focus on Wind Booster design in this work, an Elliptical Savonius is used. Table 1 and Figure 3 present the final values and final design of our Savonius design. The selection of these dimensions has the purpose of installing the VAWT on the roof of the city buildings and produce a certain amount of on-site renewable energy.

**Figure 4:**
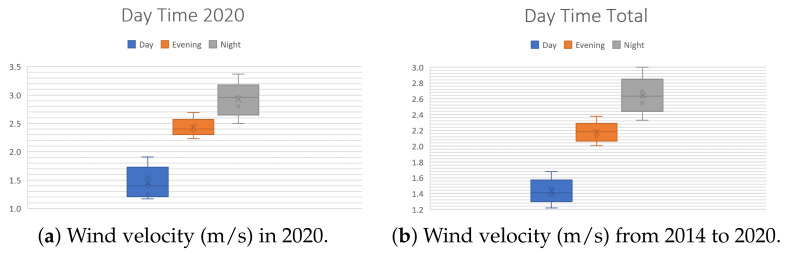
Wind velocity (m/s) for the three periods of the day.

**Figure 5:**
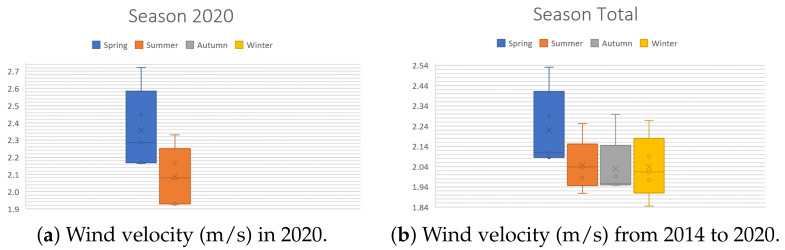
Wind velocity (m/s) for the seasons of the day.

**Figure 6:**
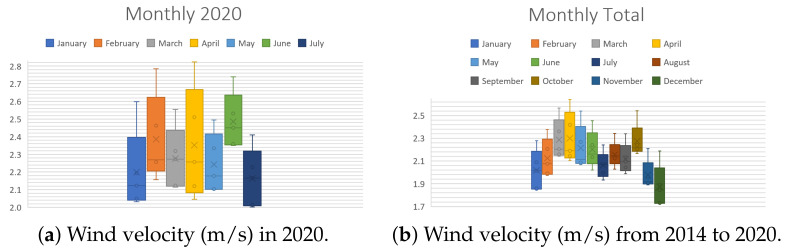
Wind velocity (m/s) for each month of the year.

In order to evaluate our design methodology, a case study is required. In order to do so, the wind conditions of Mexico City were selected for analysis: the monitoring data from 1 January 2014 to 18 July 2020 [38] were used to extract the following information about the wind speed in Mexico City. The data were obtained from the 25 meteorological stations that measure this variable 24/7. The aspects to study are the following:

- **Periods of the day**. There are three periods per day: morning (1:00 a.m. to 9:00 a.m.), afternoon (9:00 a.m. to 4:00 p.m.) and night (4:00 p.m. to 12:00 p.m.). These are useful to analyze the changes in wind speed throughout the day. Figure 4 illustrates the behavior of the wind velocity (m/s) during the periods of the day. Note in Figure 4b that during nights, the wind has a maximum speed of 3 m/s.
- **Seasons of the year**: spring (21 March to 20 June), summer (21 June to 20 September), autumn (21 September to 20 December), winter (21 December to 20 March). These are useful to observe how the climatic changes in each season affect the speed of this phenomenon. Figure 5 shows the behavior of the wind velocity (m/s) during different seasons. Figure 5a displays only that of spring and summer since it is currently mid-2020.
- **Months of the year**. To understand how wind speed varies during the 12 divisions of a year, Figure 6 exhibits that the months with lower wind velocity are January, February, July, August, September, November, and December. Contrastingly, March, April, May, June, and October have more significant velocities. Figure 6a shows only values from January to July since it is currently mid-2020.

Finally, averaging the values of all the stations during the indicated period, the average is 2.04 m/s, so the Wind Booster’s design uses this value. The database and the rest of the graphs are available in [39].

### 2.2. Select an Initial Wind Boosters and Choose the Best One

Based on the state of the art, this section proposes three different Wind Booster models. SolidWorks®is used to generate the 3D model and fluid analysis. Following our case study, the presented models are based on the previously calculated wind velocity of 2.04 m/s; however, this methodology can be applied to any city for which the wind condition measurements are available. For SolidWorks configuration, see Appendix A.

**Figure 7:**
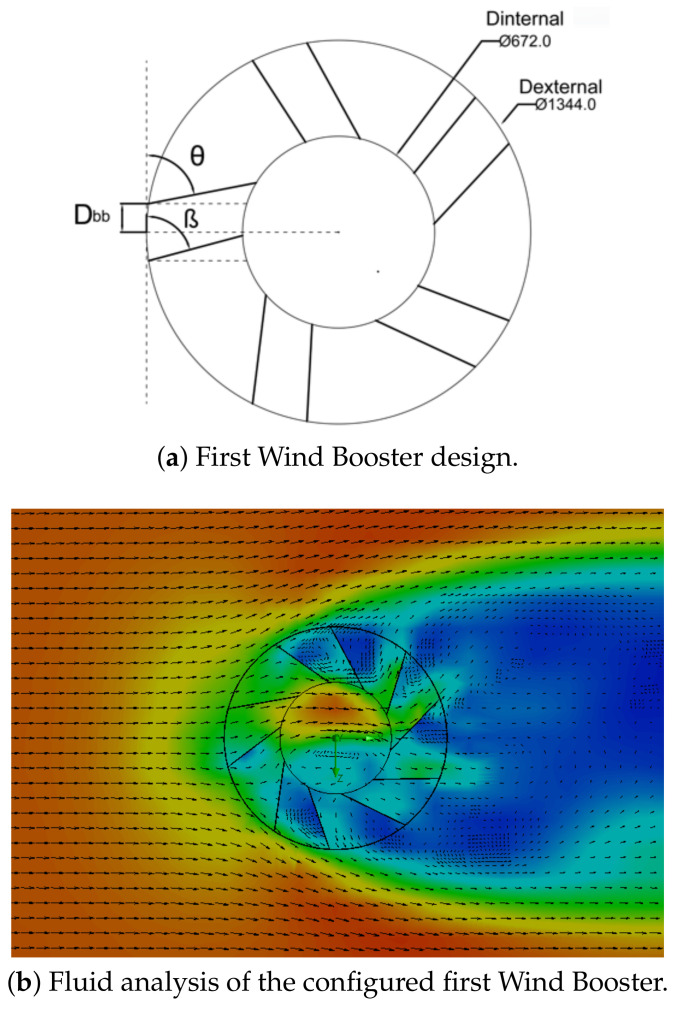
First Wind Booster design.

**Figure 8:**
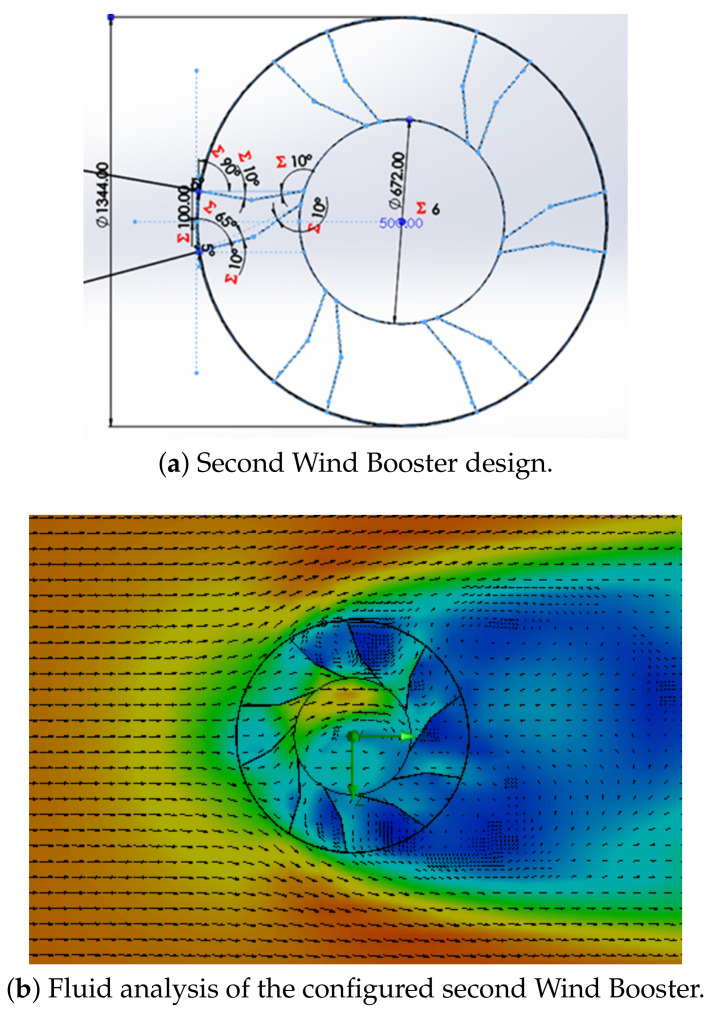
Second Wind Booster design.

**Figure 9:**
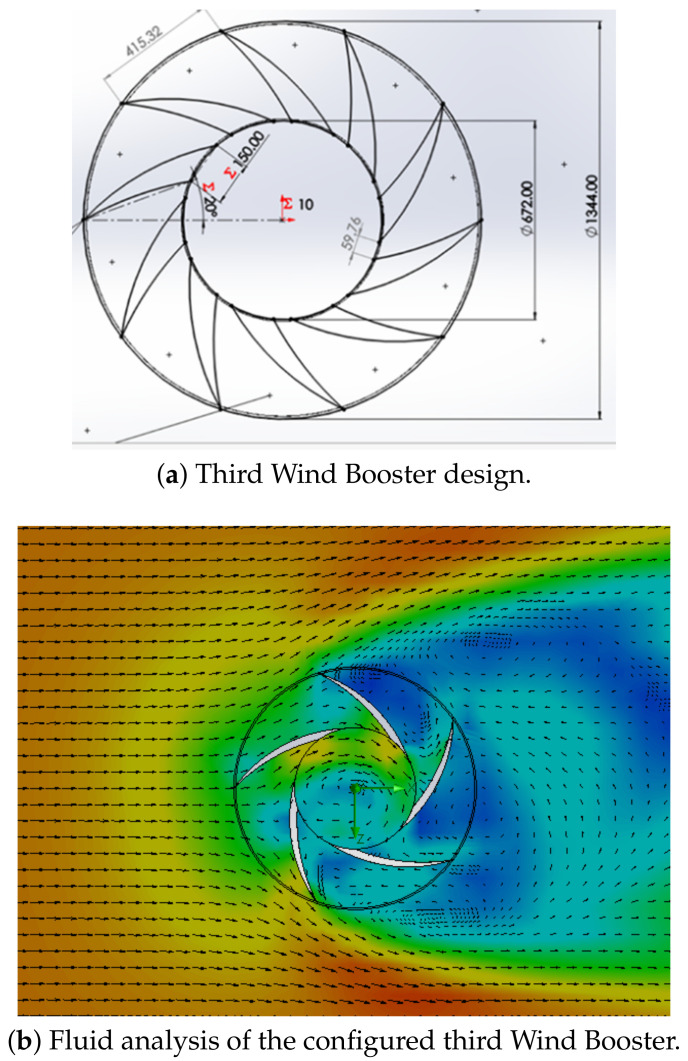
Third Wind Booster design.

1. **Wind Booster with pairs of straight blades**. The purpose of this design is to analyze the behavior of the ODGV with these kinds of bends. Figure 7 shows an initial configuration of this Wind Booster that has the following values: the number of pairs of blades ($N_{pb}$) = 4, internal diameter ($D_{internal}$) = 672 mm, external diameter ($D_{external}$) = 1344 mm, distance between the blades ($D_{bb}$) = 100 mm, angle of the odd blade (θ) = $75^{\circ }$, angle of the pair blade (β) = $79^{\circ }$. After some tests, for the fluid analysis, values that are changed are, $\theta =74^{\circ }$, $\beta =50^{\circ }$, and $D_{bb}=150$ mm. As seen in the upper internal part in Figure 7b, the ODGV lets the maximum wind speed pass, visualized in orange.
2. **Wind Booster with pairs of bent blades**. This model incorporates an intermediate angle in the blades. The idea is to manage the wind flow with more aerodynamicity. Figure 8 shows the initial configuration of this Wind Booster that has the following values: bent angle (α) = $10^{\circ }$, $N_{pb}=6$, $D_{internal}=672$ mm, $D_{external}=1344$ mm, $D_{bb}=100$ mm, $\theta =100^{\circ }$, $\beta =50^{\circ }$. After some experiments, for the fluid analysis, the values that are changed are $\alpha =9.9^{\circ }$, $D_{bb}=175$ mm, $N_{pb}=5$, $\theta =80^{\circ }$, $\beta =53^{\circ }$. Notice that in Figure 8b, the internal wind velocity is lower than in Figure 7b.
3. **Wind Booster with curved blades**. The curved style uses the theory of fluid dynamics that indicates that a curved profile is more efficient than a straight one. The concept of pairs of blades is not necessary. Figure 9a shows the starting configuration of this Wind Booster that has the next values, number of blades ($N_{b}$) = 10, blade base width ($B_{bw}$) = 150 mm, blade tilt angle ($B_{ta}$) = $20^{\circ }$. After several experiments, for the fluid analysis, the new values are $N_{b}=5$, $B_{bw}=180$ mm, $B_{ta}=29.8^{\circ }$. Figure 9b shows an increase in speed; however, the maximum wind speed is not found inside the wind booster, showing that it does not help as much as desired.

From these three proposed designs, the first one produces a higher speed inside the Wind Booster. Therefore, it is selected for the next part of the process. The variables to be optimized are θ, β, and $D_{bb}$.

### 2.3. Design of Experiments

**Figure 10:**
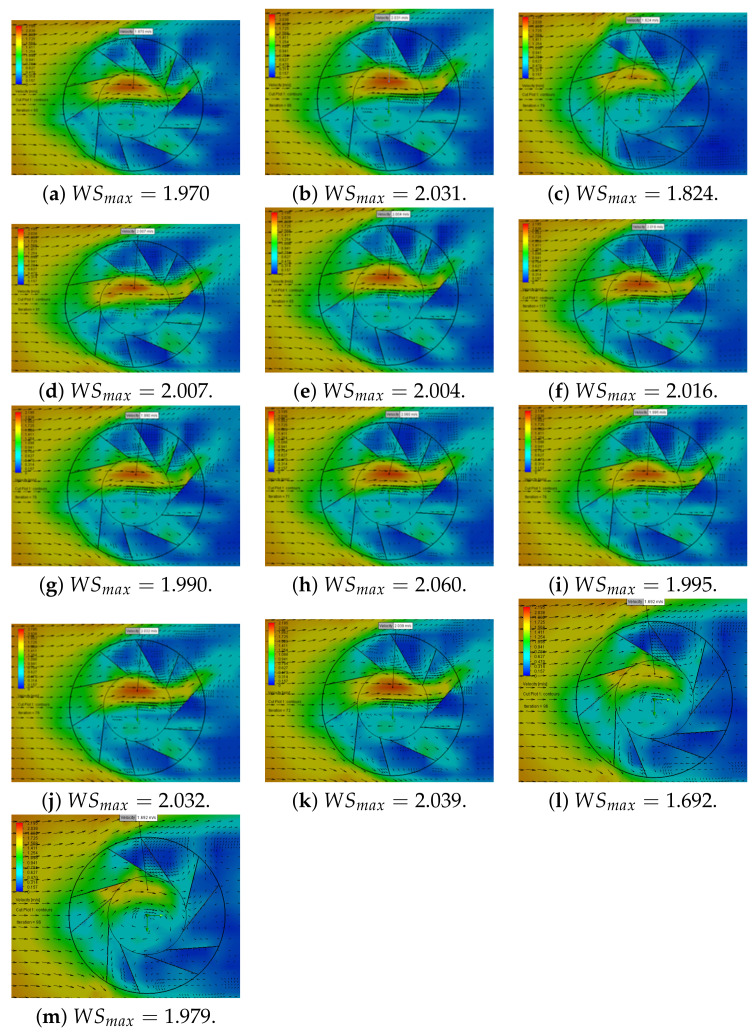
Results of flow simulations under bisection DOE.

**Figure 11:**
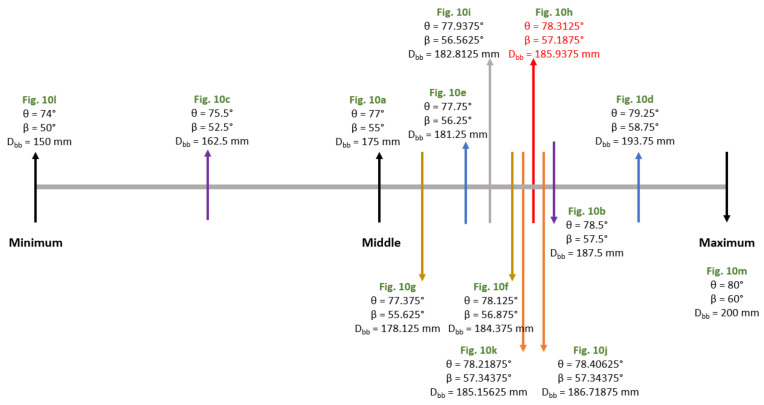
Evolution of the Wind Booster configuration using the bisection algorithm.

Design of experiments (DOE) is a systematical method to establish the connection between factors affecting a process with the output of that process; this information is used to handle process inputs to optimize the output [40]. Following this, we considered two DOE methods in the literature: factorial of two levels [40] and bisection [41], of which the latter yielded better results for this work in particular. This subsection includes all the steps in the light red color in Figure 2. Bisection begins with the selection of the input variables and the range of each one. For our case, these are as follows: $\theta_{max}=80^{\circ }$, $\theta_{min}$ $=74^{\circ }$, $\beta_{max}$ $=60^{\circ }$, $\beta_{min}$ $=50^{\circ }$, $D_{bb-max}$ =200 mm, $D_{bb-min}$ =150 mm. Table 2 shows the values used for each used Wind Booster configuration. Then, applying this method, Figure 10 shows the resulting experiments with maximum wind speed ($WS_{max}$) values. In these Figures, we measure the wind velocity inside of the Wind Booster ($WS_{max}$). The blue color indicates low speeds and the red color high speeds. Notice that Figure 10h exhibits the best results (big red zone), and the numerical values used in each configuration are shown in Figure 11.

Figure 11 presents how the Wind Booster configuration changes using the bisection algorithm to obtain the maximum wind velocity inside the ODGV. The red line indicates the best result, which corresponds to a value of $\theta =78.3125^{\circ }$, $\beta =57.1875^{\circ }$ and distance between the blades $D_{bb}=185.9397$ mm.

### 2.4. Optimization Criteria

There are two finalization criteria to stop the optimization process in Figure 2, reach a maximum of iterations, or notice a balanced improvement in wind behavior within the Wind Booster, relating speed to the amount of wind. In the case study, we reached the second criterion, as seen in Figure 10h.

## 3. Validation

**Figure 12:**
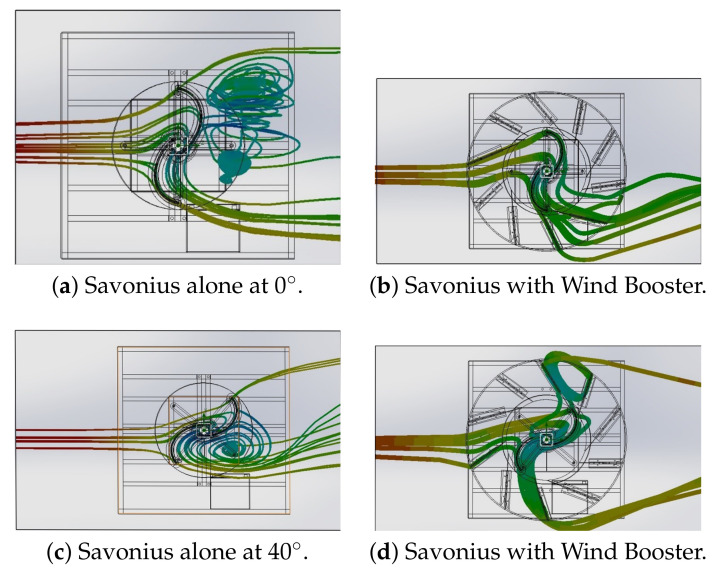
Wind Flow behavior at 2.04 m/s.

**Figure 13:**
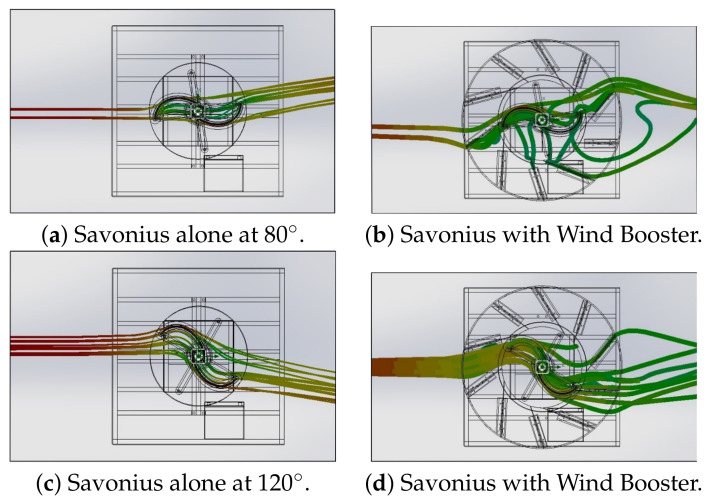
Wind Flow behavior at 2.04 m/s.

As a final step, in this section, an analysis of the performance of the Wind Booster optimized following the procedure detailed in Section 2.3 is presented. First, flow analysis of the Savonius alone versus Savonius with the Wind Booster is compared. The results of this analysis are shown in Figure 12 and Figure 13; models and more figures are available in [39]. The left side of Figure 12 shows how the wind flow goes through the Savonius surfaces. On the right side, the wind flow changes its behavior in two ways: (a) the turbulence generated after the wind impacts the Savonius disappears, and (b) the wind velocity inside the Wind Booster increases.

**Figure 14:**
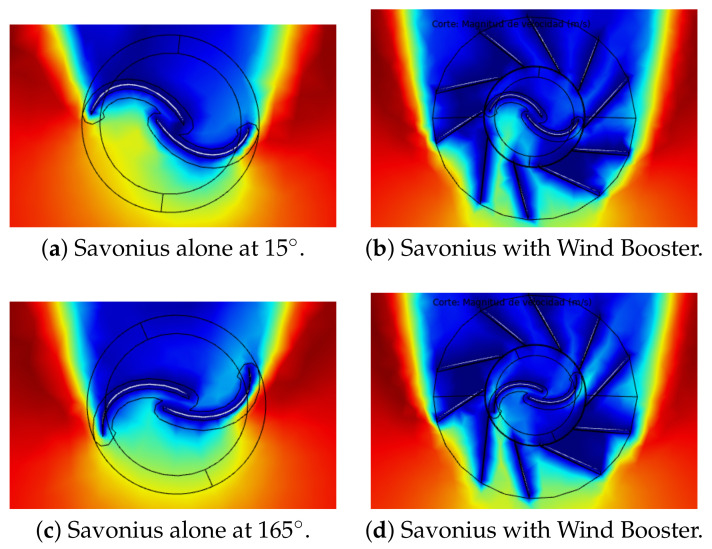
Wind flow behavior at 2.04 m/s.

Second, using Ansys Comsol Multiphysics®, a torque analysis was carried out; see configuration in Appendix B. Figure 14 illustrates the wind flow behavior, where the blue color means 0 m/s and red color 2.04 m/s. On the left side, the simple Savonius is shown. Observe that the returning blade is in contact with some wind flow, reducing the total torque of the VAWT. On the right side, the Wind Boosters help increase the wind flow and block the wind flow toward the returning blade. The total torque of the Savonius alone is 1.224 Nm and with the Wind Booster is 1.3828 Nm, resulting in a 35.23% increase in torque. Configuration files and more figures from this analysis are available in [39].

**Figure 15:**
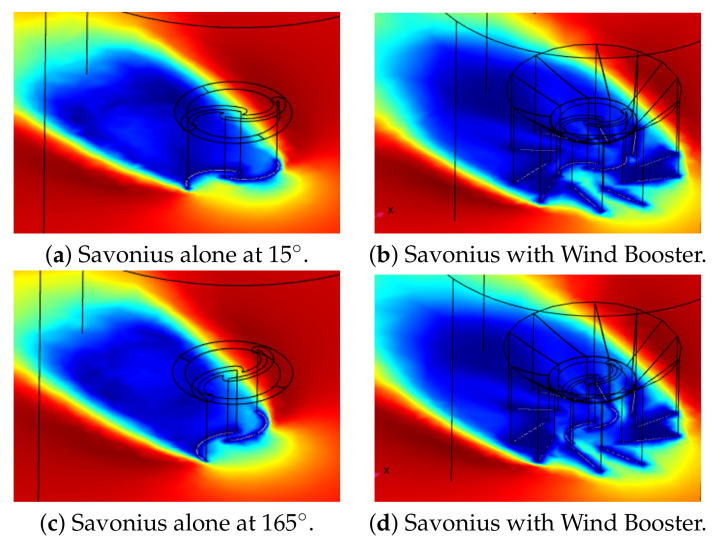
Three-dimensional view of wind flow behavior at 2.04 m/s.

Figure 15 presents a 3D view of the wind flow; notice that the Savonius with the Wind Booster receives a faster wind flow in the advancing blade while avoiding flow in the returning blade.

**Figure 16:**
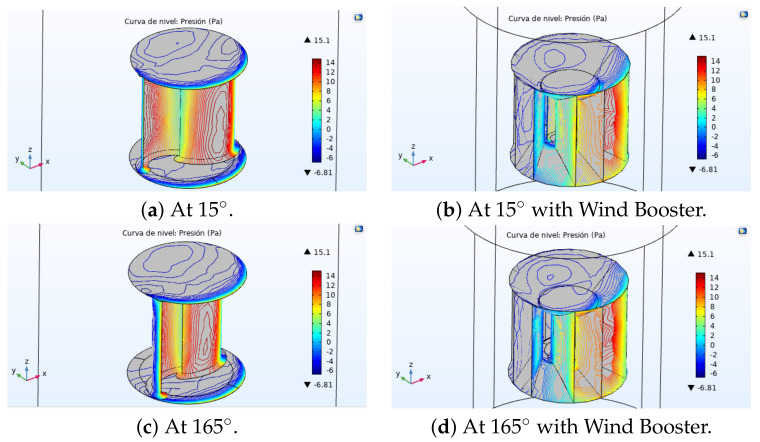
Amount of pressure that wind produces on blades. Savonius alone (**a**,**b**), and with Wind Booster (**c**,**d**).

An analysis of the amount of pressure that wind produces on each blade is presented in Figure 16. Images on the left show the Savonius alone at $15^{\circ }$ and $165^{\circ }$, and those on the right show it with the Wind Booster.

**Figure 17:**
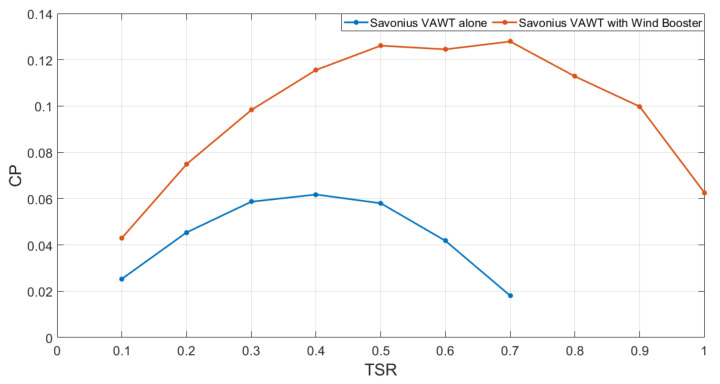
Performance improvement of the VAWT with the designed Wind Booster.

A final validation of our design is presented in Figure 17, a contrast between the CP values for different TSR values for the VAWT alone (blue line) against the VAWT with Wind Booster. Notice the notable increment of CP values, which clearly indicates the relevance of adding a Wind Booster for low-wind velocities with only using a small amount of space.

### Discussion

**Figure 18:**
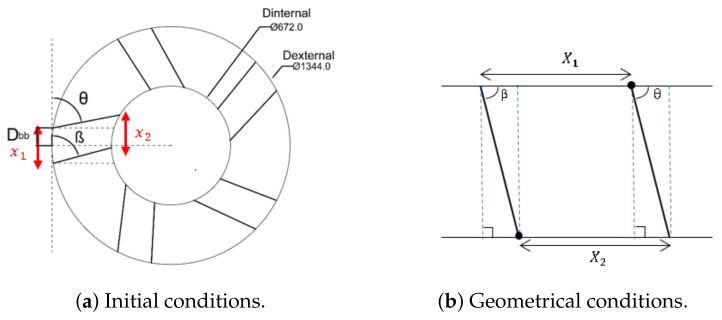
Parameter conditions.

After completing the design stage, it is interesting to highlight the following points:

1. The study of the state of the art was highly helpful in choosing the Savonius and the Wind Booster configuration, as it provided us with a starting point based on past studies.
2. To further improve the wind turbine’s performance, it is possible to apply our methodology to the Savonius and maybe other components of the wind turbine.
3. Concerning the designed variables θ, β, and $D_{bb}$, our final analysis is as follows:
  - Since the purpose is to orient the wind to the forward blade of the Savonius, the geometric limit for theta and beta is $90^{\circ }$.
  - Regarding the diameter of the Savonius, the minimum value of θ, $\theta_{min}$ has a cosecant relationship.
  - Considering the internal diameter and external diameter of the Wind Booster, we have the following relationship:
    (1) $$\theta_{min}=90^{\circ }-sin^{-1}\left(\frac{672}{1344}\right)\approx 60^{\circ }$$
    As shown in Figure 18a, the blade corresponding to that angle is not centered. Therefore, there is a correction factor of $8.4702^{\circ }$. Thus, $\theta_{min}>68.4702^{\circ }$. Following the same logic, we obtain $\beta_{min}>51.5298^{\circ }$. These calculations consider $D_{bb}=100$; any change in this variable influences each angle’s lower and upper limits.
  - We continue with the premise of *increasing the speed inside the wind booster*; for this reason, the objective is to increase speed with a decrease in the cross-sectional area. This objective leads to geometric restrictions in the design of the Wind Booster, as can be seen in Figure 18b: $X_{1}$, and $X_{2}$ must meet that $X_{1}>X_{2}$ in order to enable a reduction in the cross-sectional area; therefore, the angle θ>β.
4. Computational fluid dynamics software enables us to venture toward a challenging design since it allows to test initial designs without wasting time and money.
5. The optimization process of the Wind Booster: There are only a very few works on this matter in the literature. Natapol Korprasertsak presented different versions of this idea [33,42,43]; Korprasertsak et al. [33] used an alternating direction technique to change the angles of the blades. However, this procedure takes too long to establish a good result and only uses one physical variable of the WB. Our proposal uses three different physical values of the WB for optimization and only requires a few steps to achieve an improved result. The main limitation of our algorithm is establishing the minimum and maximum values of the variables correctly. We solved this issue with a geometrical analysis.

## 4. Conclusions

This paper presented a Wind Booster selection and optimization method, consisting of first performing an analysis of wind behavior in a certain region; then, given this behavior, experimenting with different wind boosters. Once one of these is selected, using bisection Design of Experiments, we find the set of optimal parameters that maximize wind speed inside the ODGV. As a case study, Mexico City was selected. Wind behavior was measured over a period of 6 years, and with this data, a Wind Booster with pairs of straight blades was selected and optimized, finding the optimal design values of $\theta =78.3125^{\circ }$, $\beta =57.1875^{\circ }$ and distance between blades $D_{bb}=185.9397$ mm. Using Ansys Comsol Multiphysics, a torque analysis reported a 35.23% increase in torque, using this Wind Booster configuration.

In brief, a list of the contributions is as follows: (1) a new methodology, presented in Figure 2, establishing a clear path to design an optimized wind turbine for diverse scenarios; (2) in Section 2.1, a step-by-step procedure to select an elliptical Savonius, based on the literature. Notice that the presented list of the design parameters is handy as a base for another VAWT design; (3) a case study based on Mexico City, with some interesting behaviors found. In Figure 4, notice that the high wind velocities appear at night. Figure 5 shows that spring is the season with more wind, and Figure 6 exhibits that April is the month with the highest wind; (4) Section 2.2 is devoted to a selection of the Wind Booster, starting with pairs of straight blades, then attempts with bent ones and, finally, attempts with curved ones. The first one is the best option since it produces a higher speed inside the Wind Booster and reduces the production costs; (5) the application of the bisection algorithm for the parametric optimization of the WB proved to be an excellent way to increase performance; and (6) a repository of all the designed models, analyses, and collected data presented in this paper [39].

As future work, the physical construction of the designed Wind Booster with the selected VAWT is planned, as well as experiments with an implementation to obtain measurements under specific conditions.

## Figures and Tables

**Table 1:** Parameters of the selected Savonius.

Design Parameters	Value
Cutting angle of ellipse	$45.7^{\circ }$
Number of blades	2
Chord length of blade	335.50 mm
Overall rotor diameter	550 mm
End plate diameter	600 mm
Blade thickness	3 mm
Blade torsion	$0^{\circ }$
Overlap distance	67.10 mm 20%
The largest radius of the blade ellipse	198 mm
The smallest radius of the blade ellipse	132 mm
Aspect ratio	0.7

**Table 2:** List of the values of the Wind Booster parameters used by the bisection algorithm.

Figure	θ	β	$D_{\mathrm{bb}}$	Figure	θ	β	$D_{\mathrm{bb}}$	Figure	θ	β	$D_{\mathrm{bb}}$
Figure 10a	$77^{\circ }$	$55^{\circ }$	175 mm	Figure 10f	$78.125^{\circ }$	$56.875^{\circ }$	184.375 mm	Figure 10j	$78.40625^{\circ }$	$57.34375^{\circ }$	186.71875 mm
Figure 10b	$78.5^{\circ }$	$57.5^{\circ }$	187 mm	Figure 10g	$77.375^{\circ }$	$55.625^{\circ }$	178.125 mm	Figure 10k	$78.21875^{\circ }$	$57.34375^{\circ }$	185.15625 mm
Figure 10c	$75.5^{\circ }$	$52.5^{\circ }$	162.5 mm	Figure 10h	$78.3125^{\circ }$	$57.1875^{\circ }$	185.9375 mm	Figure 10l	$74^{\circ }$	$50^{\circ }$	150 mm
Figure 10d	$79.25^{\circ }$	$58.75^{\circ }$	193.75 mm	Figure 10i	$77.9375^{\circ }$	$56.5625^{\circ }$	182.8125 mm	Figure 10m	$80^{\circ }$	$60^{\circ }$	200 mm
Figure 10e	$77.75^{\circ }$	$56.25^{\circ }$	181.25 mm								

**Figure A1:**
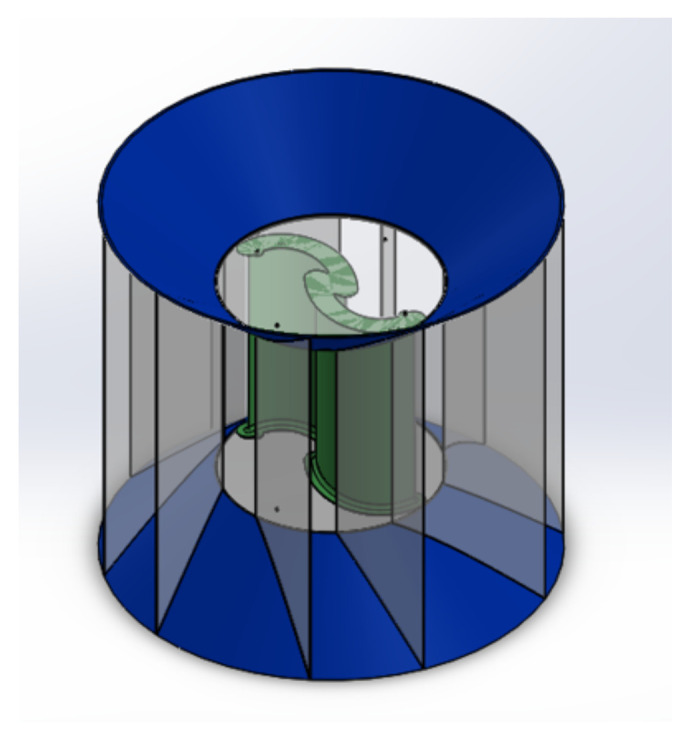
Model loaded in SolidWorks.

**Figure A2:**
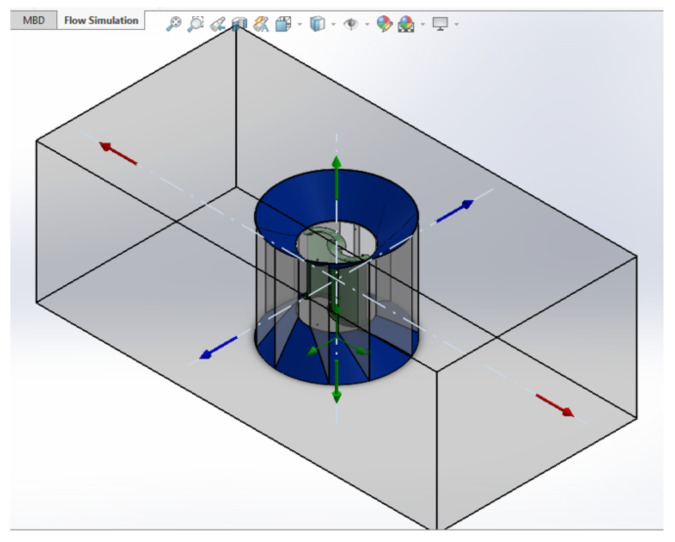
Configuration of the computational domain.

**Figure A3:**
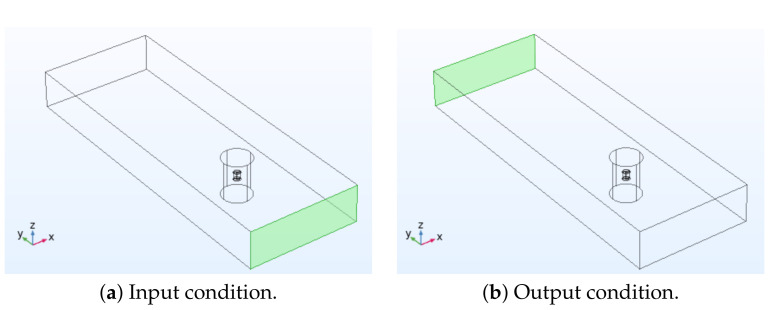
Boundary conditions.

**Figure A4:**
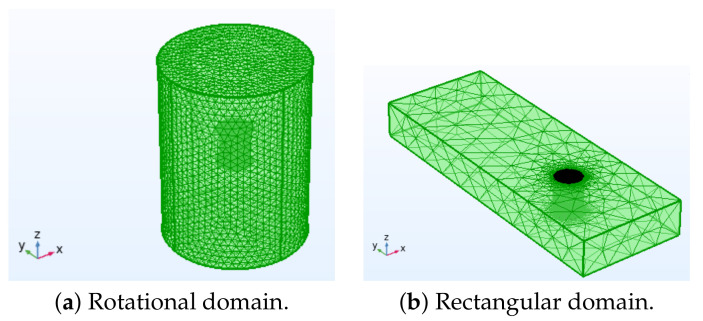
Wind Tunnel Mesh.

**Figure A5:**
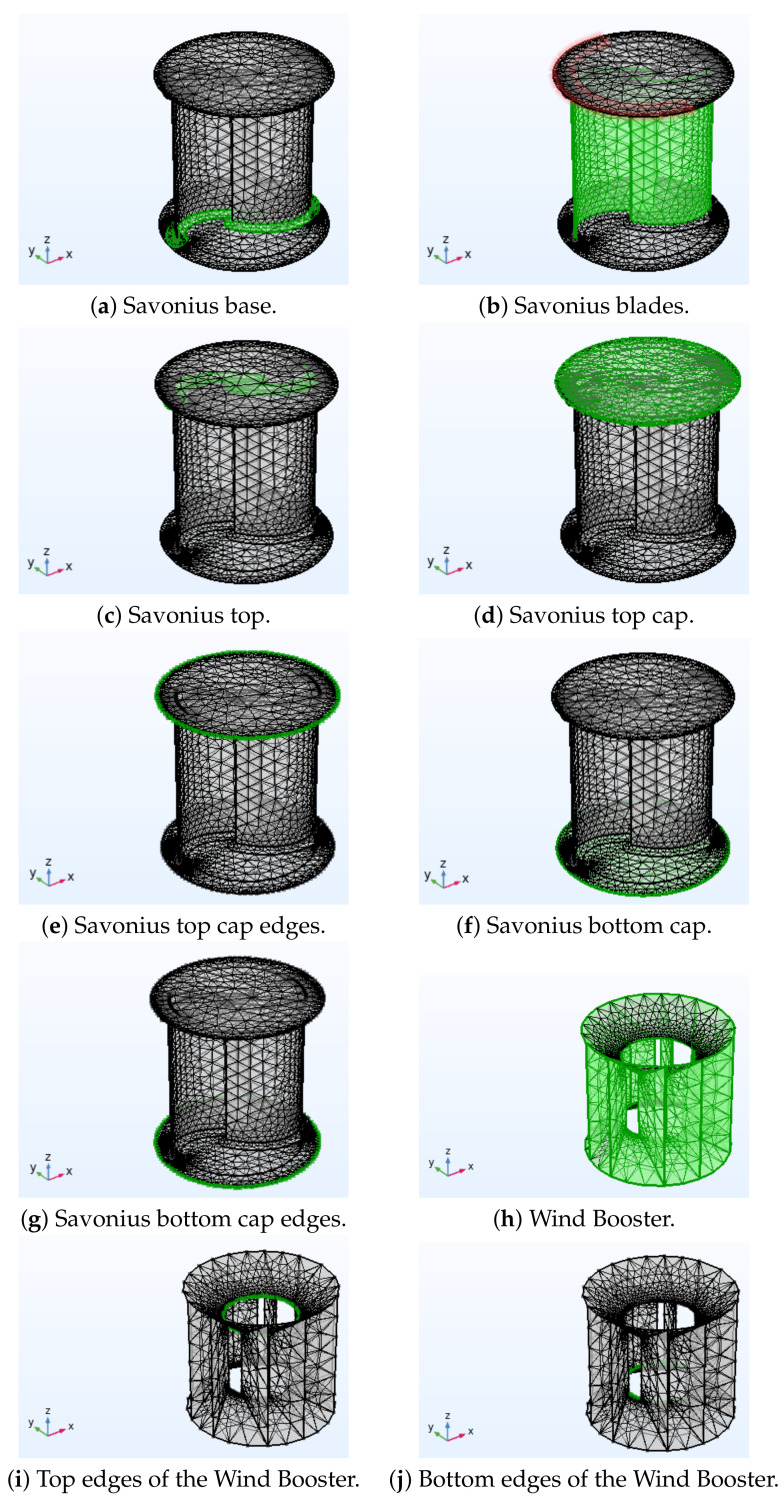
Wind turbine mesh.

## Data Availability

A repository of all the designed models, analyses, and collected data presented in this paper are in [39].

## Appendix A. SolidWorks Simulations Configuration

In this appendix, we explain the main steps to reproduce the results reported in Figure 7, Figure 8, Figure 9 and Figure 10, Figure 12 and Figure 13. The files for the Savonius and Wind Booster models can be found in the repository [39].

1. **Load the file:** Please select the desired model and load it in SolidWorks, as seen in Figure A1.
2. **Use the Wizard:** Launch the flow simulation plugin in SolidWorks and run the Wizard. Select the following options:
  - Give the project a name and leave a comment, click next.
  - Select the SI Unit system and click next.
  - Select external analysis and click next.
  - As the fluid, select air, and place in fluid type, laminar and turbulent. Do not select humidity and the number of flows; click next.
  - Use a roughness of 0 micrometers and thus represent a completely smooth surface; click next.
  - Now, assign values for pressure, temperature, wind direction, and wind speed. In our case, we use 101700 PA, 294.15 K, x-axis, 2.04 m/s respectively. Click finish.
3. **Computational domain:** Before starting the simulation, it is necessary to assign values to the computational domain’s three axes. Select each axis with the mouse to set a value as seen in Figure A2. It is advisable not to use ample space; otherwise, the simulation will take a long time.

## Appendix B. COMSOL Simulations Configuration

1. **Computational Domain**
  *Air:* The study contemplates a computational domain to simulate a wind tunnel in which is immersed the wind turbine. We divide this domain into two subdomains, the first being larger and shaped like a rectangular prism, and the second with a cylindrical shape called the rotational domain, whose center coincides with the center of the geometry of the wind turbine; see Table A1.
  **Table A1:** Dimensions of the wind computational domains.
  Domain	Size (mm)	Position (mm)
  Rectangular domain	*(9000 × 25,000 × 3000)* (*x,y,z*)	*(0 × 5000 × 500)* (*x,y,z*)
  Rotational domain	*(1160,3000)**(Radio,Altura)*	*(0,0,−1000)**(x,y,z)*
  *Wind turbine:* The rest of each study’s computational domains correspond to the different pieces that make up the Savonius wind turbine assembly itself and the Wind Booster. Their parameters and dimensions are detailed in the CAD files of each one. They can be grouped into two sets of domains called the wind turbine domain and the booster domain, respectively. The method to finalize the geometry is Form Union.
2. **To repair the geometries**, we use diverse virtual operations, such as deleting holes, collapsing faces, and composing faces, among others. As a result, we obtain more uniform and cleaner domains that simplify the meshing process.
3. **Materials:** We only consider two materials—air (we use the default settings) and for the solids, we use PLA plastic defined in Table A2.
  **Table A2:** Physical properties of PLA plastic.
  Propierty	Valor	Unit
  Density	1252	kg/m${}^{3}$
  Poisson’s ratio	0.36	–
  Young’s modulus	1.28 × 10${}^{9}$	Pa
4. **Physics:** The physics contemplated in the study is fluid dynamics. In particular, we simulate a single-phase and laminar fluid flow without considering any turbulence model. This physical model only applies to computational wind domains, and we use all initial properties and conditions default values.
  *Boundary conditions.* (a) Input: We take the wind tunnel domain’s front face as the fluid inlet with a constant speed. We highlight in green this face, as shown in Figure A3a; (b) Output: As an output condition, we consider the face opposite the wind tunnel inlet with a relative pressure of 0 Pa taken at the fluid outlet (see Figure A3b).
  *Symmetry and wall:* Finally, as symmetry conditions, the wind tunnel domain’s remaining faces are taken, and only the domains corresponding to the wind turbine and the wind booster are considered wall conditions.
5. **Mesh:** We carry out the meshing process in separate parts: building the mesh for the wind turbine domains and building the mesh for the wind domains.
  *Wind tunnel:* We use a tetrahedral mesh as shown in Figure A4 with its corresponding conditions in Table A3.
  **Table A3:** Mesh parameters.
  Figure	Calibrated	Preset Size	Maximum Element Size	Minimum Element Size
  Figure A4a	Fluid dynamics	Extremely Fine	120 (mm)	1 (mm)
  Figure A4b	Fluid dynamics	Extremely thick	Predefined	120 (mm)
  *Wind turbine:* We use a triangular mesh as shown in Figure A5 with its corresponding conditions in Table A4.
  **Table A4:** Mesh parameters.
  Figure	Calibrated	Preset Size	Maximum Element Size	Minimum Element Size
  Figure A5a	General Physics	Normal	Predefined	25 (mm)
  Figure A5b	General Physics	Extremely fine	Predefined	Predefined
  Figure A5c	General Physics	Normal	Predefined	25 (mm)
  Figure A5d *	General Physics	Normal	Predefined	165 (mm)
  Figure A5f **	General Physics	Normal	Predefined	185 (mm)
  Figure A5h ***	General Physics	Fine	Predefined	Predefined
  * Additionally, an element distribution is configured with a finite number of elements adjacent to the selected edge in Figure A5e, setting the number of elements to 50; ** Additionally, an element distribution is configured with a finite number of elements adjacent to the selected edge in Figure A5g, setting the number of elements to 50; *** Additionally, an element distribution is configured with a finite number of elements adjacent to the selected edge in Figure A5i,j, setting the number of elements to 10.

## References

[1] Canseco M. (2010). Energías Renovables en América Latina.

[2] WWEA (2020). World Wind Capacity at 650.8 GW, Corona Crisis will Slow Down Markets in 2020, Renewables To Be Core of Economic Stimulus Programmes. https://wwindea.org/blog/2020/04/16/world-wind-capacity-at-650-gw/.

[3] Mexican Government (2018). PROSEDEN. Programa de Desarrollo del Sistema Eléctrico Nacional 2018–2032. Executive Summary. https://base.energia.gob.mx/prodesen/PRODESEN2018/EXECUTIVE_SUMMARY_PRODESEN_2018-2032.pdf.

[4] Chong W., Naghavi M., Poh S., Mahlia T., Pan K. (2011). Techno-economic analysis of a wind—Solar hybrid renewable energy system with rainwater collection feature for urban high-rise application. Appl. Energy.

[5] Chong W., Fazlizan A., Poh S., Pan K., Hew W., Hsiao F. (2013). The design, simulation and testing of an urban vertical axis wind turbine with the omni-direction-guide-vane. Appl. Energy.

[6] Heo Y.G., Choi N.J., Choi K.H., Ji H.S., Kim K.C. (2016). CFD study on aerodynamic power output of a 110 kW building augmented wind turbine. Energy Build..

[7] Haase Matthias L.E. (2015). Building Augmented Wind Turbines—BAWT: Integrated Solutions and Technologies of Small Wind Turbines. http://hdl.handle.net/11250/2388620.

[8] Zhu H., Li C., Hao W., Ding Q., Yu W. (2018). Investigation on aerodynamic characteristics of building augmented vertical axis wind turbine. J. Renew. Sustain. Energy.

[9] Casini M. (2016). Small vertical axis wind turbines for energy efficiency of buildings. J. Clean Energy Technol..

[10] Kazak J.K., Świąder M. (2018). SOLIS—A Novel Decision Support Tool for the Assessment of Solar Radiation in ArcGIS. Energies.

[11] Vilar A.Á., Xydis G., Nanaki E.A. (2020). Small Wind: A Review of Challenges and Opportunities. Sustaining Resources for Tomorrow.

[12] Deshmukh A. (2009). The Role of Decentralized Renewable Energy for Rural Electrification. Maharashtra Case Study, India. Master’s Thesis.

[13] Martin J. (2009). Distributed vs. Centralized Electricity Generation: Are We Witnessing a Change of Paradigm. https://www.vernimmen.net/ftp/An_introduction_to_distributed_generation.pdf.

[14] Möllerström E., Ottermo F., Hylander J., Bernhoff H. (2016). Noise emission of a 200 kW vertical axis wind turbine. Energies.

[15] Hau E. (2013). Wind Turbines: Fundamentals, Technologies, Application, Economics.

[16] Akwa J.V., Vielmo H.A., Petry A.P. (2012). A review on the performance of Savonius wind turbines. Renew. Sustain. Energy Rev..

[17] Savonius S.J. (1931). The S-rotor and its applications. Mech. Eng..

[18] Ogawa T., Yoshida H., Yokota Y. (1989). Development of Rotational Speed Control Systems for a Savonius-Type Wind Turbine. ASME J. Fluids Eng..

[19] Grinspan A., Saha U. (2005). Experimental investigation of twisted bladed Savonius wind turbine rotor. Int. Energy J..

[20] Kacprzak K., Liskiewicz G., Sobczak K. (2013). Numerical investigation of conventional and modified Savonius wind turbines. Renew. Energy.

[21] Roy S., Mukherjee P., Saha U.K. (2014). Aerodynamic performance evaluation of a novel Savonius-style wind turbine under an oriented jet. Gas Turbine India Conference.

[22] Alom N., Kolaparthi S.C., Gadde S.C., Saha U.K. (2016). Aerodynamic design optimization of elliptical-bladed Savonius-style wind turbine by numerical simulations. International Conference on Offshore Mechanics and Arctic Engineering.

[23] Mari M., Venturini M., Beyene A. (2017). A novel geometry for vertical axis wind turbines based on the savonius concept. J. Energy Resour. Technol..

[24] Piskorz W. (2012). Wind Turbine with Cylindrical Rotor. Patent.

[25] Piskorz W., Piskorz T., Piskorz I. (2016). Multisegment Vertical Axis Wind Turbine. U.S. Patent.

[26] Wong K.H., Chong W.T., Sukiman N.L., Poh S.C., Shiah Y.C., Wang C.T. (2017). Performance enhancements on vertical axis wind turbines using flow augmentation systems: A review. Renew. Sustain. Energy Rev..

[27] Jamieson P. (2018). Innovation in Wind Turbine Design.

[28] Roy S., Saha U.K. (2013). Review of experimental investigations into the design, performance and optimization of the Savonius rotor. Proc. Inst. Mech. Eng. Part A J. Power Energy.

[29] Golecha K., Eldho T., Prabhu S. (2011). Influence of the deflector plate on the performance of modified Savonius water turbine. Appl. Energy.

[30] Altan B.D., Atılgan M., Özdamar A. (2008). An experimental study on improvement of a Savonius rotor performance with curtaining. Exp. Therm. Fluid Sci..

[31] Chong W., Pan K., Poh S., Fazlizan A., Oon C., Badarudin A., Nik-Ghazali N. (2013). Performance investigation of a power augmented vertical axis wind turbine for urban high-rise application. Renew. Energy.

[32] Pope K., Rodrigues V., Doyle R., Tsopelas A., Gravelsins R., Naterer G., Tsang E. (2010). Effects of stator vanes on power coefficients of a zephyr vertical axis wind turbine. Renew. Energy.

[33] Korprasertsak N., Leephakpreeda T. (2016). Analysis and optimal design of wind boosters for Vertical Axis Wind Turbines at low wind speed. J. Wind. Eng. Ind. Aerodyn..

[34] Wong K.H., Chong W., Yap H., Fazlizan A., Omar W., Poh S., Hsiao F. (2014). The design and flow simulation of a power-augmented shroud for urban wind turbine system. Energy Procedia.

[35] Nobile R., Vahdati M., Barlow J.F., Mewburn-Crook A. (2014). Unsteady flow simulation of a vertical axis augmented wind turbine: A two-dimensional study. J. Wind. Eng. Ind. Aerodyn..

[36] Manwell J.F., McGowan J.G., Rogers A.L. (2010). Wind Energy Explained: Theory, Design and Application.

[37] Alom N., Saha U.K. (2018). Four decades of research into the augmentation techniques of Savonius wind turbine rotor. J. Energy Resour. Technol..

[38] Mexico City’s Government (2020). Air Quality (in Spanish: *Calidad del aire*). http://www.aire.cdmx.gob.mx/.

[39] Moreno-Armendáriz M.A., Duchanoy C.A., Calvo H., Salcedo-Castañeda J.S., Ayala-Canseco M., Ibarra-Ontiveros E., García D. WindBooster Data. https://github.com/Duchanoy/WindBooster_Data.

[40] Goupy J., Creighton L. (2007). Introduction to Design of Experiments with JMP Examples.

[41] Atkinson K.E. (2008). An Introduction to Numerical Analysis.

[42] Korprasertsak N. (2015). CFD-Based Analysis and Optimization of Wind Boosters for Low Speed Vertical Axis Wind Turbines. Ph.D. Thesis.

[43] Korprasertsak N., Leephakpreeda T. (2015). Optimal design of wind boosters for low speed vertical axis wind turbines. Appl. Mech. Mater..

